# Predictive value of invasive mechanical ventilation parameters for mortality in COVID-19 related ARDS: a retrospective cohort study

**DOI:** 10.1038/s41598-024-64725-x

**Published:** 2024-06-14

**Authors:** Luis Gutiérrez, Karina Araya, Mara Becerra, Camilo Pérez, Jorge Valenzuela, Lydia Lera, Pablo A. Lizana, Mariano del Sol, Rodrigo Muñoz-Cofré

**Affiliations:** 1 Servicio de Medicina Física y Rehabilitación, Hospital El Carmen de Maipú, Camino A Rinconada 1201, Santiago, Chile; 2 Latin Division, Keiser University eCampus, Fort Lauderdale, FL USA; 3 Laboratory of Epidemiology and Morphological Sciences, Instituto de Biología, Pontificia Universidad Católica de Valparaíso, Valparaíso, Chile; 4 Programa de Doctorado en Ciencias Morfológicas, Universidad de La Frontera, Temuco, Chile

**Keywords:** COVID-19, Mechanical Ventilation, Survival Analyses, Diseases, Health care

## Abstract

The 2019 coronavirus (COVID-19) can generate acute respiratory distress syndrome (ARDS), requiring advanced management within the Intensive Care Unit (ICU) using invasive mechanical ventilation (IMV However, managing this phenomenon has seen learning and improvements through direct experience. Therefore, this study aims were to describe the assessment of the different IMV variables in patients with post-COVID-19 hospitalized in the ICU and their relation with mortality. Observational and retrospective study. The sample was divided into two, the surviving group (SG) and the non-surviving group (NSG). Clinical data were extracted from the electronic clinical file and the respiratory therapist record sheet. The following information was obtained: Patient medical history: gender, age, co-morbidities, arterial gases, days on IMV, and IMV parameters. Out of a total of 101 patients, the total mortality was 32%. There was a significant decrease in respiratory rate (RR) (29.12 ± 4.24–26.78 ± 3.59, *p* = 0.006), Driving pressure (DP) (11.33 ± 2.39–9.67 ± 1.84, *p* = 0.002), Ventilatory rate (VR) (2.26 ± 0.66–1.89 ± 0.45, *p* = 0.001) and a significant rise in Static compliance (Cest) (35.49 ± 8.64–41.45 ± 9.62, *p* = 0.003) and relation between Arterial oxygen pressure/Inspirated oxygen fraction (PaO_2_/FiO_2_) (201.5 ± 53.98- 227.8 ± 52.11, *p* = 0.008) after 72 h of IMV, within the NSG compared to the SG. Apart from these points, multi-morbidity (HR = 3.208, p = 0.010) and DP (HR = 1.228, *p* = 0.030) and VR variables (HR = 2.267, *p* = 0.027) had more death probabilities. The results of this study indicate that there was a significant increase in RR, DP, VR, and CO_2_ and a significant drop in Cest and PaO_2_/FiO_2_ among the NSG compared with the SG. Apart from this, the DP and VR variables, multi-morbidity and being male. have more possibility of death.

## Introduction

A percentage of patients who were infected with COVID-19 could become gravely ill and develop acute respiratory distress syndrome (ARDS), requiring management within an Intensive Care Unit (ICU). This can lead to high risk of remaining within the unit, and multi-system complications^1–3^. While invasive mechanical ventilation (IMV) has been a primary solution for treating patients entering ICUs for COVID-19^2,3^, it did not always resolve the problem, since a number of patients connected to IMV did not survive.

The most common assessment upon initiating IMV is the “protective strategy”^4^. Lung protection strategies in IMV are directly related to reducing the harmful effects of positive pressure ventilation, among which the following stand out: barotrauma, volutrauma, atelectrauma, and biotrauma. In this sense, protective ventilation is based on reducing alveolar overdistension, maintaining an average plateau pressure < 29–30 cmH_2_Oand a Driving Pressure (DP) < 15 cmH_2_O in the airways^4^. However, these concepts come from experiences accumulated during management of classic ARDS^3,4^. Whether this body of knowledge was effective in managing ARDS arising from COVID -19 is a question which is being resolved. In this sense, assessment and exhaustive monitoring of IMV variables is fundamental^5^.

Gattinoni et al., (2020) affirmed that one of the fundamental differences between habitual respiratory pathologies and post-COVID-19 ARDS treated with IMV was the high compliance observed in patients connected to IMV for COVID-19 due to the vascular component, which turned out to be independent of the extent of the damage^6^On the other hand, Möhlenkamp et al. (2020) report that worsening inflammation may indicate a transition to lungs with high elastance, low distensibility, higher recruitment capacity, and response to a PEEP, i.e., a typical ARDS^7^. Spinelli et al., (2021) showed uncertainty about management of post-COVID-19 ARDS, which considers the gas measurement parameter, specifically the relation between Arterial oxygen pressure/Inspirated oxygen fraction (PaO_2_/FiO_2_), as the only guideline for clinical decision making^8^.

There are variables such as gender, age, and chronic non-communicable diseases that, when interacting with COVID-19, have a higher risk of developing and experiencing progression to more severe disease states. Also, older patients with chronic non-communicable diseases have a higher rate of intensive care unit (ICU) admission and mortality due to COVID-19^9,10^.

The programming of IMV in ICU patients with ARDS due to COVID-19 is challenging because its evolution is heterogeneous and does not always behave like conventional ARDS; this clinical behavior demands optimal monitoring. In addition, the variability of morbid antecedents must be considered because they are non-modifiable factors at the time of admission to the ICU. Therefore, this study aims were to describe the assessment of the different IMV variables in patients with post-COVID-19 hospitalized in the ICU and their relation with mortality.

## Methodos

The present study is observational and retrospective. It was done in the ICU of Hospital El Carmen de Maipú (HEC), Chile. HEC is a public hospital with 500 beds. The ICU has 48 beds, which were all used to care for COVID-19 patients during the pandemic.

### Study population

**Figure 1:**
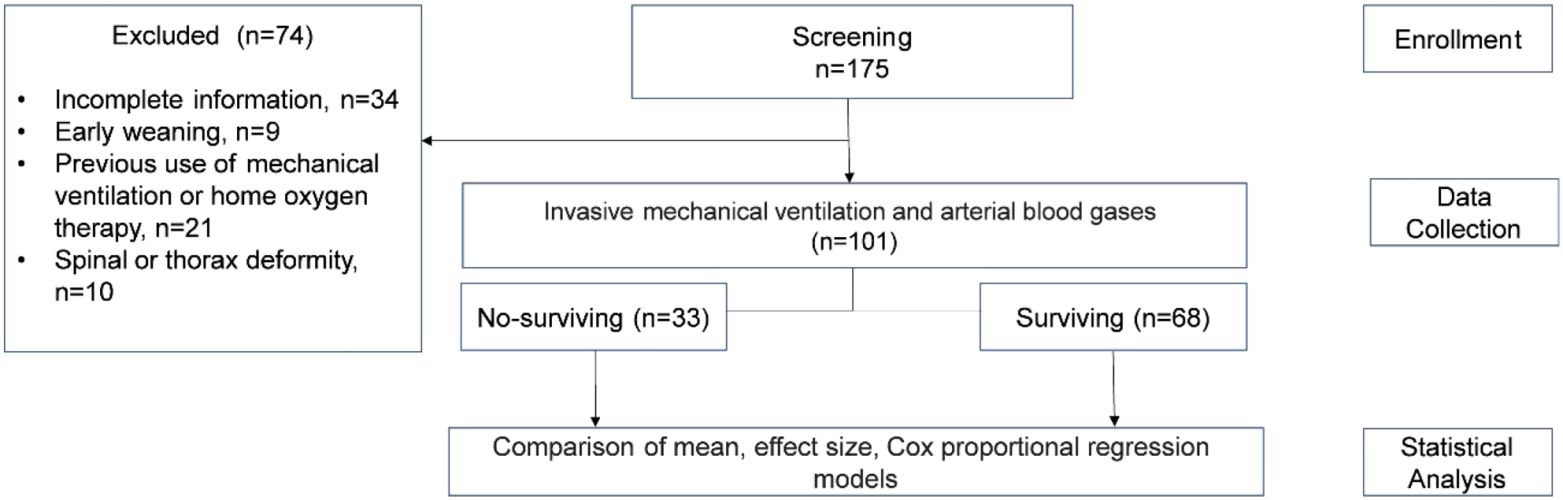
Flowcharts of the studied sample.

Patients with ARDS due to COVID-19 from March to September 2021 were evaluated. The sample was divided into two groups: surviving (SG) and non-surviving (NSG) (Fig. 1). The inclusion criteria were being hospitalized in the HEC ICU, being over 18 years old, having a SDRA for COVID-19 diagnosis (confirmed with PCR [ +] and thorax scanner), being connected to IMV for more than 72 h and having sedation-analgesia required for a *sedation agitation scale* score of 1.The exclusion criteria were: musculoskeletal disorders in the spinal column and/or thorax, home IMV users, and patients who began weaning and/or remained in pressure-controlled modes in the first 72 h. The criteria were adjusted according to the recommendations of WHO (2020), Botta et al., 2021, and Torres et al., 2021, on the management of patients with moderate-severe ARDS caused by COVID-19^1,3,11^. This study was done according to the Ethics Code of the World Medical Association (Helsinki Declaration) for experiments with human beings and was approved by the Scientific Ethics Committee of the Central Metropolitan Health Service (392/2021).

### Data gathering

Patients were followed up until they were discharged from the unit. All clinical data were extracted from the electronic clinical chart (Florence, clinical version 19.3) and respiratory therapist records. The following data were obtained: Patient health background; gender, age, comorbidities, Arterial Gases, days on IMV, APACHE (calculated at the admission of the ICU pre-connection to the IMV) and the following IMV parameters: Positive End Expiratory Pressure (PEEP) in cmH_2_O, Tidal Volume (TV) in mL, Respiratory Rate (RR) in rpm, Peak Pressure (Pp) in cmH_2_O, Pressure Plateau (Ppl) in cmH_2_O, Flow en L/min, Static compliance (Cest) in ml/cmH_2_O, Driving Pressure (DP) in cmH_2_O, Mechanical Power (MP) in J/min [following the formula from Cressoni et al.^12^: *(0.098) × (FR×Vt) × (Peak pressure—½ DP)*], Ventilatory Ratio (VR) [as per the formula in Sinha et al. ^13^: *Volume per minute measured × Pco*_*2*_* measured/ Volume per minute predicted × ideal Pco*_*2*_], PaO_2_/FiO_2_ and CO_2_. These parameters were obtained after 24 and 72 h of connection to IMV, as per the reports from Botta et al.^1^, Serpa Neto et al.^14^ and Sinha et al.^13^.

### Statistical analysis

The programs used to perform statistical analysis were SPSS version 28.0.1.1. and STATA15 (StataCorp.2015. Stata Statistical Software, Release 14. College Station, TX, StataCorp LP). Descriptive variable management was done via mean ± standard deviation and median, as appropriate. The effect size (ES) was calculated with Cohen’s d (values < 0.2 indicated a small effect size, 0.5 medium, and 0.8 indicated a high-magnitude effect). Data normality was determined with the Kolmogorov–Smirnov test. The difference between SG and NSG was defined with the Student’s T-test or Mann–Whitney U. The differences between the SG and NSG at 24 and 72 h were defined with the Student’s T-test or Wilcoxon test.

Cox proportional regression models for 2–68 days of mortality were estimated to analyze adjusted hazard ratios (HR) and 95% confidence interval (CI) of death by the presence of multimorbidity and “ventilation reduction”. No violations of the proportional hazard assumptions were detected. For the final model, a backward test was done to generate a parsimonious model. The variables were dichotomized as follows: PEEP ≥ 12, Ppl ≥ 23, Cest < 48, DP ≥ 12, MP ≥ 17, VR ≥ 2 and PaO_2_/FiO_2_ ≥ 200. Statistical significance was established at a value of p < 0.05. The incidence density was also calculated.

### Ethics approval and consent to participate

This study was done according to the Ethics Code of the World Medical Association (Helsinki Declaration) for experiments with human beings and was approved by the Scientific Ethics Committee of the Central Metropolitan Health Service (392/2021). All participants and/or their legal guardian(s) provided written informed consent to participate in the investigation.

## Results

Out of 175 patients, 101 were included while 74 were excluded, 34 due to incomplete information, 9 by early weaning, 21 by Previous use of mechanical ventilation or home oxygen therapy and 10 by spinal or thorax deformity (Fig. 1). Among the 101 subjects, the average age was 58 ± 13 years, and 41 patients were female. Median IMV connection was 15 days, and the APACHE obtained was 22 ± 4 points. Mortality was 32%. The most common chronic diseases were hypertension, diabetes, and obesity at 46%, 26% and 22% respectively. 30% of patients had tracheostomies [Table 1].

**Table 1:** Demographic and morbid characteristics of the recruited patients.

	Total (n = 101)	Non-Surviving (n = 33)	Surviving (n = 68)	p value
Age (years)	58 ± 13	62 ± 10	55 ± 14	0.021^t^
Gender (F/M)	41/60	10/23	31/37	
APACHE (points)	22.79 ± 4.31	24.39 ± 5.11	22.01 ± 3.67	0.008^t^
Obesity (n/%)	22 (21)	5 (15)	17 (25)	0.312χ
Disease (n/%)				
COPD	10 (9)	5 (15)	5 (7)	0.288^χ^
Diabetes mellitus	26 (25)	9 (27)	17 (25)	0.812^χ^
Neurological	6 (5)	3 (9)	3 (4)	0.389^χ^
Heart	2 (1)	1 (3)	1 (1.5)	0.548^χ^
Renal	4 (3)	1 (3)	3 (4)	0.100^χ^
Hypertension	47 (46)	20 (60)	27 (39)	0.05^χ^
Tracheostomy (n/%)	31 (30)	12 (36)	19 (27)	0.490^χ^
Days IMV	9 (15–25)	16 (20–25)	8 (13–25)	0.005^ MW^

*F* female, *M* male, *n* number, *COPD* chronic obstructive pulmonary disease, *IMV* invasive mechanical ventilation, χ Chi squared, *t* Student’s T-test, *MW* Mann–Whitney U test.

The average age in the SG was 55 ± 14 years, and 31 patients were female. Median IMV connection was 13 days, and the APACHE obtained was 24 ± 5 points. The most common chronic comorbidities were hypertension, obesity, and diabetes at 39%, 25% and 25% respectively. In addition, 27% of patients were tracheostomized [Table 1]. Within the NSG, the average age was 62 ± 10 years, and 10 patients were female. Median IMV connection was 20 days, and the APACHE score was 22 ± 3 points. The most common chronic diseases were hypertension, diabetes, and obesity at 60%, 27%, and 15% respectively. 36% of patients were tracheostomized [Table 1].

When comparing measurements for IMV and arterial blood gases (ABG) between the SG and NSG at 24 h, statistical differences arose in the following areas: Cest (35.36 ± 8.52 ml/cmH_2_O v/s 39.66 ± 9.62 ml/cmH_2_O;* p* = 0.03, respectively), DP (11.06 ± 2.45 cmH_2_O v/s 10 ± 2.11 cmH_2_O; *p* = 0.02, respectively) and PaO_2_/FiO_2_ (189.3 ± 57.05 v/s 197 ± 57.86; *p* = 0.0001, respectively) [Table 2].

**Table 2:** Comparison of the invasive mechanical ventilation variables at 24 h between the non-survivor group and the survivor group.

	NSG (n = 33)	CI 95%	SG (n = 68)	CI 95%	p value
PEEP (cmH_2_O)	11.55 ± 2.13	(10.79–12.30)	11.46 ± 2.21	(10.92–11.99)	0.672^ MW^
Tv (ml x kg)	377.3 ± 48.04	(360.2–394.3)	379.9 ± 51.79	(367.3–392.4)	0.606^ MW^
RR (rpm)	28.5 ± 3.64	(27.25–29.84)	27.21 ± 3.22	(26.43–27.99)	0.157^ MW^
Peak pressure (cmH_2_O)	26.91 ± 2.68	(25.96–27.86)	25.85 ± 3.86	(24.92–26.79)	0.070^ MW^
Plateau pressure (cmH_2_O)	22.61 ± 2.47	(21.73–23.48)	21.46 ± 3.36	(20.64–22.27)	0.072^ MW^
Flow (L/min)	48.5 ± 11.03	(44.63–52.46)	45.18 ± 10.62	(42.61–47.75)	0.143^t^
Cest (ml/cmH_2_O)	35.36 ± 8.52	(32.33–38.38)	39.66 ± 9.62	(37.33–41.99)	0.030^t^
DP (cmH_2_O)	11.06 ± 2.45	(10.34–11.79)	10.00 ± 2.11	(9.48–10.51)	0.022^ MW^
MP (J/min)	21.64 ± 4.97	(19.88–23.41)	19.80 ± 6.00	(18.35–21.25)	0.130^ MW^
VR	2.15 ± 0.55	(1.96–2.35)	1.95 ± 0.57	(1.81–2.35)	0.088^ MW^
CO_2_ (mmHg)	46.18 ± 9.74	(42.73–49.64)	43.21 ± 8.70	(41.10–45.31)	0.780^ MW^
PaO_2_/FiO_2_	189.30 ± 57.05	(169.0–209.5)	197.0 ± 57.86	(183.0–211.0)	0.0001^t^

*SG* surviving group, *NSG* non-surviving group, *CI* confidence interval, *PEEP* Positive end-expiratory pressure, *Tv* tidal volume, *RR* respiratory rate, *Cest* Static compliance, *DP* driving pressure, *MP* mechanical power, *VR* ventilatory ratio, *CO*_*2*_ Carbon dioxide, *PaO*_*2*_*/FiO*_*2*_ Arterial oxygen pressure/Inspirated oxygen fraction, *t* Student’s T-test, *MW* Mann–Whitney U test.

Comparing measurements of IMV and ABG parameters between the NSG and SG after 72 h presented statistical differences in: RR (29.12 ± 4.24 v/s GS 26.78 ± 3.59; *p* = 0.006, respectively), Ppl (22.3 ± 2.67 cmH_2_O v/s 20.22 ± 2.93 cmH_2_O; *p* = 0.001, respectively), Cest (35.49 ± 8.64 ml/cmH_2_O v/s 41.45 ± 9.62 ml/cmH_2_O; *p* = 0.003, respectively), DP (11.33 ± 2.39 cmH_2_O v/s 9.67 ± 1.84 cmH_2_O; *p* = 0.002, respectively), VR (2.26 ± 0.66 v/s 1.89 ± 0.45; *p* = 0.001, respectively), CO_2_ (45.91 ± 8.59 mmHg v/s 41.9 ± 6.4 mmHg; *p* = 0.009, respectively) and PaO_2_/FiO_2_ (201.5 ± 53.98 v/s 227.8 ± 52.11; *p* = 0.008, respectively) [Table 3].

**Table 3:** Comparison of the invasive mechanical ventilation variables at 72 h between the non-survivor group and the survivor group.

	NSG (n = 33)	CI 95%	SG (n = 68)	CI 95%	p value
PEEP (cmH_2_O)	10.97 ± 2.31	(10.15–11.79)	10.54 ± 2.38	(9.96–11.12)	0.545^ MW^
Tv (ml x kg)	385.6 ± 50.86	(367.6–403.6)	387.4 ± 51.86	(374.8–399.9)	0.703^ MW^
RR (rpm)	29.12 ± 4.24	(27.61–30.63)	26.78 ± 3.59	(25.91–27.65)	0.006^ MW^
Peak pressure (cmH_2_O)	26.33 ± 3.47	(25.10–27.56)	24.99 ± 4.35	(23.93–26.04)	0.064^ MW^
Plateau pressure (cmH_2_O)	22.30 ± 2.67	(21.35–23.25)	20.22 ± 2.93	(19.51–20.93)	0.001^ MW^
Flow (L/min)	48.09 ± 12.38	(43.70–52.48)	44.41 ± 12.33	(41.43–47.40)	0.163^t^
Cest (ml/cmH_2_O)	35.49 ± 8.64	(32.43–38.56)	41.45 ± 9.62	(39.12–43.78)	0.003^t^
DP (cmH_2_O)	11.33 ± 2.39	(10.48–12.18)	9.67 ± 1.84	(9.23–10.12)	0.002^ MW^
MP (J/min)	22.77 ± 5.76	(20.73–24.82)	20.86 ± 7.3	(19.09–22.63)	0.190^t^
VR	2.26 ± 0.66	(2.02–2.49)	1.89 ± 0.45	(1.78–2.00)	0.001^t^
CO_2_ (mmHg)	45.91 ± 8.59	(42.86–48.96)	41.90 ± 6.4	(40.34–43.45)	0.009^t^
PaO_2_/FiO_2_	201.5 ± 53.98	(182.4–220.7)	227.8 ± 52.11	(215.2–240.4)	0.008^ MW^

*SG* surviving group, *NSG* non-surviving group, *CI* confidence interval, *PEEP* positive end-expiratory pressure, *Tv* tidal volume, *RR* respiratory rate, *Cest* static compliance, *DP* driving pressure, *MP* mechanical power, *VR* ventilatory ratio, *CO*_2_, carbon dioxide, *PaO*_2_/*FiO*_2_ arterial oxygen pressure/inspirated oxygen fraction, *t* student’s T-test, *MW* Mann–Whitney U test.

After analyzing the SG, a significant decrease was observed in PEEP (ES = 0.218), Pp and Ppl (ES = 0.392) between 24 and 72 h (*p* = 0.006; *p* = 0.0002; *p* = 0.001, respectively). In turn, PaO_2_/FiO_2_ (ES = 0.559) rose significantly (197.0 to 227.8; *p* = 0.001) [Table 4]. Analysis of the NSG showed no significant differences in the IMV and GSA parameters between 24 and 72 h [Table 5].

**Table 4:** Comparison between 24 and 72 h of the variables of invasive mechanical ventilation in the surviving group.

	24 h	72 h	ES	p value
PEEP (cmH_2_O)	11.46 ± 2.21	10.54 ± 2.38	0.218	0.001^W^
Tv (ml x kg)	379.9 ± 51.79	387.4 ± 51.86	0.156	0.111^W^
RR (rpm)	27.21 ± 3.22	26.78 ± 3.59	0.333	0.174^W^
Peak pressure (cmH_2_O)	25.85 ± 3.86	24.99 ± 4.35	0.002	0.0062^W^
Plateau pressure (cmH_2_O)	21.46 ± 3.36	20.22 ± 2.93	0.392	0.0002^W^
Flow (L/min)	45.18 ± 10.62	44.41 ± 12.33	0.089	0.439^W^
Cest(ml/cmH_2_O)	39.66 ± 9.62	41.45 ± 9.62	0.214	0.07^t^
DP (cmH_2_O)	10.00 ± 2.11	9.67 ± 1.84	0.632	0.112^W^
MP (J/min)	19.80 ± 6.00	20.86 ± 7.3	0.153	0.857^W^
VR	1.95 ± 0.57	1.89 ± 0.45	0.062	0.549^W^
CO_2_ (mmHg)	43.21 ± 8.70	41.90 ± 6.40	0.171	0.263^W^
PaO_2_/FiO_2_	197.0 ± 57.86	227.8 ± 52.11	0.559	0.0001^W^

*ES* effect size *dCohen*, *PEEP* positive end-expiratory pressure, *Tv* tidal volume, *RR* respiratory rate, *Cest* static compliance, *DP* driving pressure, *MP* mechanical power, *VR* ventilatory ratio, *CO*_2_ carbon dioxide, *PaO*_2_/*FiO*_2_ arterial oxygen pressure/Inspirated oxygen fraction, *t* student’s T-test, *W* Wilcoxon.

**Table 5:** Comparison between 24 and 72 h of the invasive mechanical ventilation variables in the non-survivor group.

	24 h	72 h	ES	p value
PEEP (cmH_2_O)	11.55 ± 2.13	10.97 ± 2.31	0.500	0.126^t^
Tv (ml x kg)	377.3 ± 48.04	385.6 ± 50.86	0.163	0.059^t^
RR (rpm)	28.5 ± 3.64	29.12 ± 4.24	0.282	0.290^W^
Peak pressure (cmH_2_O)	26.91 ± 2.68	26.33 ± 3.47	0	0.275^t^
Plateau pressure (cmH_2_O)	22.61 ± 2.47	22.30 ± 2.67	0	0.638^W^
Flow (L/min)	48.5 ± 11.03	48.09 ± 12.38	0	0.782^t^
Cest (ml/cmH_2_O)	35.36 ± 8.52	35.49 ± 8.64	0	0.908^t^
DP (cmH_2_O)	11.06 ± 2.45	11.33 ± 2.39	0	0.392^W^
MP (J/min)	21.64 ± 4.97	22.77 ± 5.76	0.200	0.329^t^
VR	2.15 ± 0.55	2.26 ± 0.66	0	0.348^W^
CO_2_ (mmHg)	46.18 ± 9.74	45.91 ± 8.59	0.021	0.839^t^
PaO_2_/FiO2	189.30 ± 57.05	201.5 ± 53.98	0.219	0.297^t^

*ES* effect size *dCohen*, *PEEP* positive end-expiratory pressure, *Tv* tidal volume, *RR* respiratory rate, *Cest* static compliance, *DP* driving pressure, *MP* mechanical power, *VR* ventilatory ratio, *CO*_2_ carbon dioxide, *PaO*_2_/*FiO*_2_ arterial oxygen pressure/Inspirated oxygen fraction, *t* student’s T-test, *W* Wilcoxon.

After adjusting for age and gender, high death risks were observed among patients with two or more chronic diseases ([HR] = 3.2, *p* = 0.01), DP cmH_2_O ([HR] = 1.2; *p* = 0.03) and VR ([HR] = 2.3, *p* = 0.03). By contrast, being a woman is a protective factor in the Cox regression model ([HR] = 0.3, *p* < 0.01). The age variable is also not significant in this model (*p* > 0.05) [Table 6].

**Table 6:** Cox regression model for mortality according to morbidity and mortality and ventilatory variables adjusted for gender and age.

	Hazard Ratio	[95% CI]	p value
Multimorbility (> 2)	3.208	1.324–7.771	0.010
Obesity	0.554	0.205–1.497	0.244
DP	1.228	1.021–1.477	0.030
VR	2.267	1.099–4.675	0.027
Age (> 60 years)	0.510	0.208–1.250	0.141
Gender (female)	0.250	0.103–0.607	0.002

Reference category of independent variables: < 2 CD; < 60 years; men; non-obese.

*CI* confidence interval, *DP* driving pressure, *VR* ventilatory ratio.

Calculation of incidence density took place after 1975 person-days of follow-up (median follow-up 5.4 days), with 33 cases (death from COVID-19) identified (incidence density rate = 1.67 per 100 persons/days).

## Discussion

The objective of the present study was to describe IMV variables and their mortality impacts for patients with COVID-19-related ARDS. The main results indicate that after 24 h, the SG showed lower DP (*p* = 0.022), as well as after 72 h. Within the same group, significant falls were observed in RR (*p* = 0.006), DP (*p* = 0.002), VR (*p* = 0.001) and Cest (*p* = 0.002) compared with the NSG. Finally, patients with two or more deadly morbid conditions, DP ≥ 12 cmH_2_O and VR ≥ 2, had greater mortality risks. In this regard, Parada-Gereda et al., 2023 reported that VR, Cest, DP, and age were identified as risk factors for 30-day mortality in patients with more than five days of ARDS IMV due to COVID-19, the findings of Parada-Gereda et al. support the results obtained in the present investigation^15^.

The present study showed that DP values were significantly lower after both 24 and 72 h in the SG compared to the NSG (10.00 ± 2.11–11.06 ± 2.45; *p* = 0.022 and 9.67 ± 1.84–11.33 ± 2.39; p = 0.002, respectively). Amato et al. 2015^16^ and Costa et al. 2021^4^, highlighted the importance of DP and its relation with mortality among ARDS patients. Botta et al., in a review about IMV management among patients with post-COVID-19 ARDS, observed that in all consulted centers DP was measured, highlighting its relation with mortality^1^. In this context, the results obtained in the present study aligned with existing evidence, given that the SG showed lower DP and values ≥ 12 cmH_2_O had a higher chance of death (HZ = 1.2; *p* = 0.03)**.** We recommend the inclusion of DP as a variable to be considered in the management and prognosis of ARDS by COVID-19 due to its ease of measurement and interpretation for the healthcare team. However, despite efforts to maintain DP below 15 cmH_2_O, a percentage of patients did not survive, so it is necessary for future research to include other variables of higher complexity, such as recruitment maneuvers or prone positions.

Costa includes RR, among other variables, in his mortality model in ARDS patients^4^. The results of the present investigation indicate that the RR at 72 h was significantly higher in the NSG compared to the SG (29.12 ± 4.24—26.78 ± 3.59; *p* = 0.006, respectively). These values coincide with improvements in gas measurement values (CO_2_: 41.90 ± 6.4 vs 45.91 ± 8.59, *p* = 0.009; SG vs NSG, respectively). This association is explained because the health team's focus is directed by CO_2_ in the blood and how it behaves with the different IMV. This could indicate that RR scheduling, contributes to improving clinical status^17^. The suggestion here is thus to move into objective RR programming systems, to clear up the experience of treating health teams.

In this study, we also considered dead space; several studies agree that VR is a valid tool to monitor dead space indirectly, providing useful information for IMV management^2,13,15^. The results of the present study indicate that VR at 72 h was significantly higher in the NSG compared to the SG (2.26 ± 0.66 vs 1.89 ± 0.45; *p* = 0.001), which meshes with extant research. For instance, Morales et al. (2019) observed ARDS patients and saw a significant rise in VR within the NSG compared with the SG (1.9 vs 1.6; *p* < 0.01)^17^. Sinha P et al. (2019) studied the clinical usefulness of VR among ARDS patients and also reported greater VR among non-surviving patients compared with the surviving group (2.02 ± 0.8 vs 1.75 ± 0.5; *p* < 0.001)^18^. Furthermore, the Cox mortality analysis indicated that patients with VR ≥ 2 had a higher probability of death (HR = 2.267; *p* = 0.01). This finding is similar to Torres et al. (2021) who reported an association between VR and mortality after 72 h of connection to IMV (OR = 1.04 [IC 1.01–1.07]; *p* = 0.030)^2^. The evidence presented so far indicates that RV measurement provides valuable information about patient mortality. VR is thus an easy monitoring index and highly useful for clinical practice^19^, so it is suggested that its measurement be performed routinely.

Regarding the Cest, also showed significant changes between the NSG and SG, both at 24 and 72 h (35.36 ml/cmH_2_O v/s 39.66 ml/cmH_2_O; *p* = 0.03 and 35.49 ml/cmH_2_O v/s 41.45 ml/cmH_2_O; *p* = 0.003 respectively). Vandenbunder et al. (2021) studied the behavior of static compliance among 372 patients with post-COVID-19 ARDS; with a significant decrease in this value on day 14 compared to day 1 of connection to IMV (37.8 ± 11.4 ml/cmH_2_O vs 31.2 ± 14.4 ml/cmH_2_O, *p* < 0.001). However, this decrease had no association with patient survival after 28 days (*p* = 0.55)^20^. Boscolo et al. (2021), carried out a multi-centered study including 241 patients. While their results did not show a linear regression between Cest and mortality, they observed that patients with a Cest below 48 ml/cmH_2_O had a higher mortality^21^. The results of this study indicated that the Cest average in both group is below 48 ml/cmH_2_O. However, there is a significant Cest decrease in GNS at 24 and 72 h with regards to GS (Tables 2 and 3). It is thus necessary to determine particular cutoff points, considering that the clinical conditions of patients connected to IMV. The usefulness of the Cest value alone regarding mortality is controversial; however, measuring Cest provides clinical information about the patient's condition and contributes to contextualizing and making decisions in each specific case.

Finally, when comparing ABGs, PaO_2_/FiO_2_ was significantly higher in the SG compared with the NSG, at both 24 and 72 h (197.0 ± 57.86 vs 189.30 ± 57.05; *p* = 0.0001 y 227.8 ± 52.11 vs 201.5 ± 53.98; *p* = 0.008, respectively). In this point, the available evidence indicates that a value for PaO_2_/FiO_2_ ≤ 200 is a mortality risk factor^22,23^. The results of the present study indicate PaO_2_/FiO_2_ ≤ 200 mmHg in the NSG, which was complemented with higher CO_2_ at 72 h (45.91 ± 8.59 mmHg vs 41.90 ± 6.4 mmHg; *p* = 0.009). In this regard, Torres et al. (2021) observed significantly higher CO_2_ values in the non-surviving group at 72 h (50.3 [44.0–58.0] vs 46.0 [40.0–51.0]; *p* < 0.001) compared with the surviving group^2^. Similarly, Hueda-Zavaleta et al., (2022) concluded in patients with COVID-19 connected to IMV that PaO_2_/FiO_2_ values ​​ < 222.5 at 24 h of IMV are associated with higher in-hospital mortality (hazard ratios = 2.87)^24^. Optimization of ABGs is one of the objectives of IMV in patients with ARDS. Together with the other indicators mentioned above (DP, VR, RR, Cest), it makes decision-making multifactorial and more detailed.

On the other hand, there are patient-specific variables. In this context, Chaturvedi et al., (2022) studied the variability of the effects of COVID-19 linked to gender. One of its main conclusions is that men have a greater risk of ICU care and mortality than women^25^. This aligns with the findings from the present study, where being female turned out to be a protective factor against mortality (Table 6). Another key point is the effect of co-morbidities on mortality among COVID-19 patients. In this regard, Gómez et al., (2021) used a multivariable model corrected by age and co-morbidities to determine that there was a significant association between being male and mortality within a cohort (odds ratio = 1.96; *p* = 0.0001)^26^. In summary, the evidence supports the results obtained, i.e., male COVID-19 patients with two or more comorbidities have a higher mortality risk (Table 6).

The present study has strengths, such as describing the assessment of respiratory mechanics of IMV among COVID-19 patients and determining variables linked with mortality; however, it also has limitations that must be indicated. As with all retrospective analyses, the lack of information at the moment of data tabulation affected the number of patients to analyze and the number of variables analyzed, since data such as neuromuscular blocking, prone position, PaO_2_/FiO_2_ before connection to IMV, and SOFA score, among others, could not be recovered. In addition, the COVID-19 pandemic led to high health team rotation, making data tracking and continuity difficult, which could impact the recording of the analyzed variables. Finally, including a control group would provide greater statistical strength to the analyses performed. Therefore, prospective studies are needed to validate the findings mentioned above.

## Conclusion

The results of this study indicate that there was a significant rise in RR, DP, VR, and CO_2_ and a significant decrease in Cest and PaO_2_/FiO_2_ among the NSG compared with the SG. Apart from this, the variables for DP, VR, multimorbidity, and male gender had higher probabilities of death. Therefore, the DP, Cest and VR variables are easily accessible and have significant clinical application during the IMV process. In any case, more prospective studies are needed to complement the data obtained in our study.

## Data Availability

The datasets used and/or analysed during the current study are available from the corresponding author on reasonable request.

## Abbreviations

ARDS: Acute respiratory distress syndrome
ICU: Intensive care unit
IMV: Invasive mechanical ventilation
SG: Surviving group
NSG: Non-surviving group
PaO_2_/FiO_2_: Arterial oxygen pressure/inspirated oxygen fraction
PEEP: Positive end expiratory pressure
TV: Tidal volume
RR: Respiratory rate
Pp: Peak pressure
Ppl: Pressure plateau
Cest: Static compliance
DP: Driving pressure
MP: Mechanical power
VR: Ventilatory ratio
CO_2_: Carbon dioxide
ABG: Arterial blood gases
